# Utility of the Red Cell Distribution Width-to-Albumin Ratio in Predicting Short-Term Mortality in Acute Exacerbations of Chronic Obstructive Pulmonary Disease: A Prospective Observational Study

**DOI:** 10.7759/cureus.99928

**Published:** 2025-12-23

**Authors:** Sai Jeevan P., Gaurav Jain, Ankit Agarwal, Nilotpal Chowdhury, Prakhar Sharma, Vaishnavi Pandith, Mayuri Gupta

**Affiliations:** 1 Department of Anaesthesiology and Critical Care, All India Institute of Medical Sciences, Rishikesh, IND; 2 Department of Pathology and Laboratory Medicine, All India Institute of Medical Sciences, Rishikesh, IND; 3 Department of Pulmonary Medicine, All India Institute of Medical Sciences, Rishikesh, IND

**Keywords:** albumin, chronic obstructive pulmonary disease, intensive care unit, mortality, red blood cell distribution width

## Abstract

Background

Acute exacerbation of chronic obstructive pulmonary disease (ECOPD) significantly contributes to intensive care unit (ICU) admissions and short-term mortality. The red cell distribution width-to-albumin ratio (RAR) has emerged as a potential biomarker for systemic inflammation and adverse outcomes. This study aimed to assess the diagnostic value of baseline RAR in predicting 28-day all-cause mortality in ICU-admitted patients with acute ECOPD.

Methodology

In a prospective, observational design, 83 consecutive patients admitted with moderate to severe ECOPD were enrolled. Baseline clinical data, laboratory parameters, and clinical severity scores were collected. Patients were followed up for 28 days. The primary outcome was 28-day all-cause mortality. Receiver operating characteristic (ROC) analysis was used to determine the baseline optimal RAR cut-off.

Results

A baseline RAR threshold of >5.43%dL/g showed excellent predictive performance for 28-day mortality (area under the receiver operating characteristic (AUROC): 0.979; sensitivity: 95.45%; specificity: 95.08%). Mortality was significantly higher in the high RAR group (87.5%) when compared to the low RAR group (1.69%) (*P* < 0.001). Kaplan-Meier survival analysis confirmed significantly improved survival in patients with lower RAR. On multivariate analysis, only baseline lactate (*P* = 0.011) and RAR (*P* = 0.008) independently predicted mortality. RAR had a poor predictive value for the failure of non-invasive ventilation.

Conclusions

Baseline RAR is a simple, accessible, and effective biomarker for predicting short-term mortality in ECOPD. Its integration into early ICU risk stratification protocols warrants further validation in larger, multi-center cohorts.

## Introduction

Chronic obstructive pulmonary disease (COPD) is a common, preventable, and treatable respiratory condition marked by persistent symptoms and airflow limitation due to airway and/or alveolar abnormalities. Despite being manageable, it remains a major global health concern, ranked as the second leading cause of death in India and fifth worldwide [1]. Acute exacerbations of COPD (ECOPD), defined as a worsening of dyspnoea, cough, or sputum within <14 days, often with tachypnoea or tachycardia, are typically triggered by infections, pollutants, or other inflammatory insults. These episodes significantly increase hospital admissions, healthcare costs, and mortality [2]. Despite advances in COPD management, early risk stratification in ECOPD, especially in ICU settings, remains challenging. Clinicians depend on clinical, radiologic, and laboratory markers, yet there is still a need for simple, cost-effective biomarkers to guide prognosis.

Red cell distribution width (RDW), reflecting variability in red blood cell size, is readily available through routine complete blood counts and has been associated with inflammation and poor outcomes in various conditions, including ECOPD. Similarly, serum albumin, a negative acute-phase reactant and nutritional marker, correlates with disease severity. Both markers have shown independent prognostic value in ECOPD [3,4]. The RDW-to-albumin ratio (RAR), which combines these two parameters, has recently emerged as a potential marker of systemic inflammation and adverse outcomes in critical illness. However, its utility in predicting short-term mortality in ICU-admitted patients with ECOPD remains under-investigated [5].

We hypothesize that RAR can help predict short-term mortality in ECOPD. This prospective observational study aims to assess the diagnostic value and optimal threshold of baseline RAR for predicting 28-day all-cause mortality in patients with ECOPD. If validated, RAR could serve as a simple, accessible tool for early risk assessment and informed clinical decision-making.

## Materials and methods

Following institutional ethical approval and written informed consent, patients aged ≥18 years, of either sex, requiring hospital admission for moderate to severe ECOPD were enrolled in a prospective observational study between May 2024 and June 2025 (CTRI/2024/05/067962). We followed ethical principles as defined by the Helsinki Declaration 2013. Moderate ECOPD was defined by the presence of at least three of the following five criteria: Dyspnea visual analog score (VAS > 5), respiratory rate > 24 breaths/minute, heart rate > 95 beats/minute, resting SaO2 < 92% breathing ambient air and/or change > 3%, C-reactive protein > 10 mg/dL, arterial blood gas (ABG) analysis showing PaO2 < 60 mmHg and/or PaCO2 > 45 mmHg but no acidosis. Severe ECOPD was defined as meeting the criteria of moderate ECOPD, along with ABG findings indicative of new onset/worsening hypercapnia and acidosis (PaCO2 > 45 mmHg and pH < 7.35). Patients with hematologic diseases, pregnancy, liver disease, diabetic ketoacidosis, acute stroke, or severe malnutrition were excluded, as they are known to independently affect either RDW or albumin levels.

Standard institutional protocols were followed for obtaining medical history, conducting clinical examinations, and managing the acute exacerbation. Clinical severity scores, including the Charlson comorbidity index (CCI), sequential organ failure assessment score (SOFA), and acute physiological and chronic health evaluation score (APACHE II), were calculated on the day of admission [6-8]. Patients were followed up for 28 days post-admission or death, whichever occurred earlier.

The primary outcome variables included RAR and the number of 28-day all-cause mortality. Venous blood samples, including RDW and serum albumin, were collected at ICU admission. RDW was measured using an automated hematology analyzer (BC 6200, Mindray, Shenzhen, China) based on the principle of electrical impedance and reported as the coefficient of variation. Serum albumin was measured spectrophotometrically upon binding to Bromocresol Green dye using an auto-analyzer (AU5800, Beckman Coulter, Brea, CA, USA). All samples were processed in a single centralized hospital laboratory, eliminating inter-laboratory variability.

The secondary outcome variables included SOFA score, APACHE II score, CCI score, hemoglobin levels, total leucocyte counts, platelet counts, lactate levels, base deficit, need for invasive mechanical ventilation (MV), time to non-invasive ventilation (NIV) failure, NIV failure rates, recurrent need for invasive MV, ICU complications, new onset bacteremia/fungemia, need for renal replacement therapy, blood or components transfusion, number of ventilator free days at 28 days post inclusion, number of vasopressor free days at 28 days post inclusion, organ failure free days (total SOFA score <6 for 24 hours) at 28 days post inclusion, length of ICU stay, length of hospital stay, ICU mortality, hospital mortality, and 28 day mortality. The outcome assessor was blinded to the RAR groups. Decisions regarding the modality of ventilation, weaning, and discharge from the ICU or hospital were based on the judgment of the treating physician.

The sample size calculation was performed using the power-ROC test function of the pROC on R software v4.1.3 (The R Foundation, Vienna, Austria). Based on pilot data indicating a 28-day all-cause mortality rate of 25% and targeting an expected area under the curve (AUC) of 0.71 [5], with RAR as the primary variable, an alpha error of 0.05, and a power of 0.8, the required sample size was calculated to be 83 patients (expected deaths = 21; expected survivors = 62).

Statistical analysis was conducted using SPSS software (version 25.0, IBM Corp., Armonk, NY) and R software (Version 4.4.0, R Foundation for Statistical Computing, Vienna, Austria). The optimal cut-off value for the RAR was determined by maximizing the sum of sensitivity and specificity (Youden Index) from the receiver operating characteristic (ROC) curve. The independent t-test and the Mann-Whitney U test were used to compare normally distributed and skewed continuous variables, respectively. Categorical variables were analyzed using the chi-square test or Fisher’s exact test, as appropriate. The impact of RAR on 28-day all-cause mortality was assessed using Kaplan-Meier survival analysis and evaluated using the log-rank test, the Grambsch-Therneau test (cox.zph function), and Cox proportional hazards (Cox PH) modeling [9]. A two-tailed *P*-value < 0.05 was considered statistically significant, with a 95% confidence interval reported.

## Results

**Figure 1:**
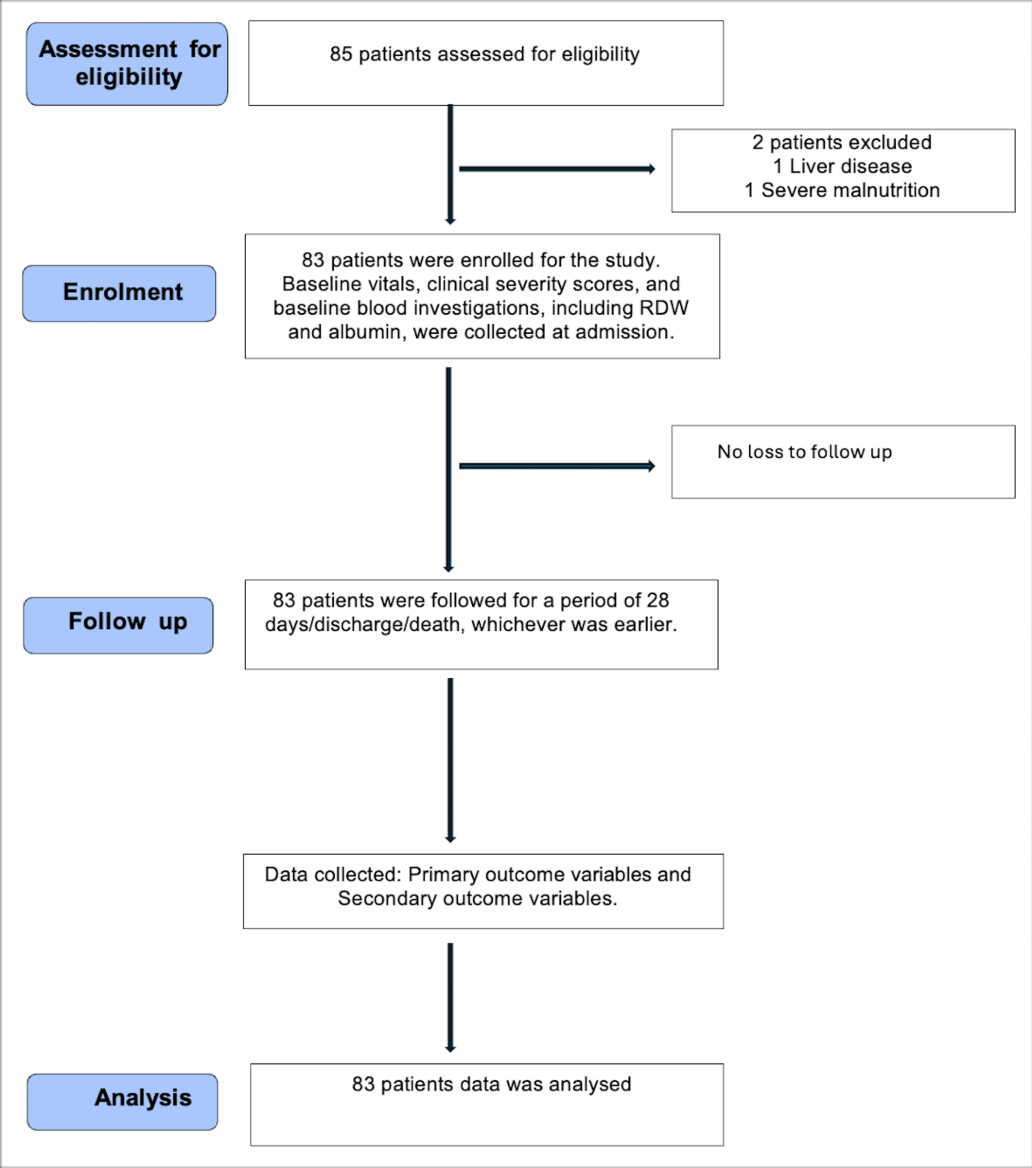
Strengthening the Reporting of Observational Studies in Epidemiology (STROBE) flow diagram of the patients studied. RDW, red cell distribution width

Of the 85 patients assessed for eligibility, 2 were excluded due to underlying liver disease and severe malnutrition. In total, 83 patients were included in the final analysis, without any dropouts (Figure 1). The cohort consisted predominantly of older adults (mean age: 58.92 years), mostly males, with an average body mass index (BMI). The predominant comorbidities were hypertension (24, 28.9%), obesity (16, 19.3%), and diabetes mellitus (9, 10.8%). The mean baseline severity scores were: APACHE II 15.58, SOFA 5.36, and CCI 1.77. The mean baseline RAR was 5.05 ± 1.35%dL/g, and 22 patients (26.5%) died within 28 days of admission (Table 1).

**Table 1:** Comparison of baseline variables between the high and low baseline RAR groups. Group 1: baseline RAR >5.43%dL/g, Group 2: baseline RAR <5.43%dL/g ***Significant at *P *< 0.05 ^$^Unpaired t test. ^€^Chi-square test. ^£^Wilcoxon-Mann-Whitney U test. All continuous parametric data presented as mean ± standard deviation (SD), continuous nonparametric data presented as median (interquartile range), categorical variables presented as frequency (percentage). RDW, red cell distribution width; RAR, red cell distribution width-to-albumin ratio; APACHE, acute physiological and chronic health evaluation; SOFA, sequential assessment of organ failure; CCI, Charlson comorbidity index

Parameter	Total (*n* = 83)	Group 1 (*n* = 24)	Group 2 (*n* = 59)	*P*-value
Age (Years)***	58.92 ± 10.40	63.13 ± 9.67	57.20 ± 10.28	0.018^$^
Gender (Male)	60 (72.3%)	19 (79.2%)	41 (69.5%)	0.372^€^
Height (cm)	160.13 ± 5.64	160.21 ± 6.99	160.10 ± 5.11	0.939^$^
Weight (kg)***	65 (60-69)	64 (55.25-65.00)	65 (60-70)	0.028^£^
Body mass index***	25.56 (23.68-26.16)	23.87 (22.66-25.60)	25.39 (24.03-27.18)	0.021^£^
Chronic kidney disease	1 (1.2%)	1 (4.16%)	0 (0%)	0.115^€^
Stroke	1 (1.2%)	1 (4.16%)	0 (0%)	0.115^€^
Coronary artery disease***	5 (6%)	5 (20.83%)	0 (0%)	<0.001^€^
Diabetes Mellitus	9 (10.8%)	2 (8.32%)	7 (11.86%)	0.639^€^
Obesity	16 (19.3%)	2 (8.32%)	14 (23.72%)	0.107^€^
Hypertension	24 (28.9%)	8 (33.33%)	16 (27.11%)	0.571^€^
Lactate***	1.8 (1.4-2.1)	2.25 (1.82-2.60)	1.60 (1.40-1.80)	<0.001^£^
Hemoglobin***	12.2 (11.40-13.40)	11.60 (10.72-12.60)	12.50 (11.60-13.40)	0.035^£^
Hematocrit***	33 (32.00-36.00)	32 (30.25-35.77)	34 (32.00-36.00)	0.041^£^
WBC count	11640 (10782-12790)	12125 (10782.5-13535)	11610 (10780-12610)	0.335^£^
Platelet count	243783.13 ± 75411.49	222833.33 ± 77756.40	252305.08 ± 74061.31	0.109^$^
RDW***	14.10 (13.20-15.10)	15.75 (14.5-16.5)	13.60 (12.9-14.3)	<0.001^£^
RAR***	4.55 (4.11-5.73)	6.91 (6.12-7.47)	4.27 (3.89-4.65)	<0.001^£^
APACHE II score***	14 (12-18)	20 (16.50-22.00)	14 (11-16)	<0.001^£^
SOFA score***	5 (4-6)	7 (5.25-10.00)	4 (4-5)	<0.001^£^
CCI score***	1 (1-2)	2 (1-3)	1 (1-2)	0.003^£^

**Figure 2:**
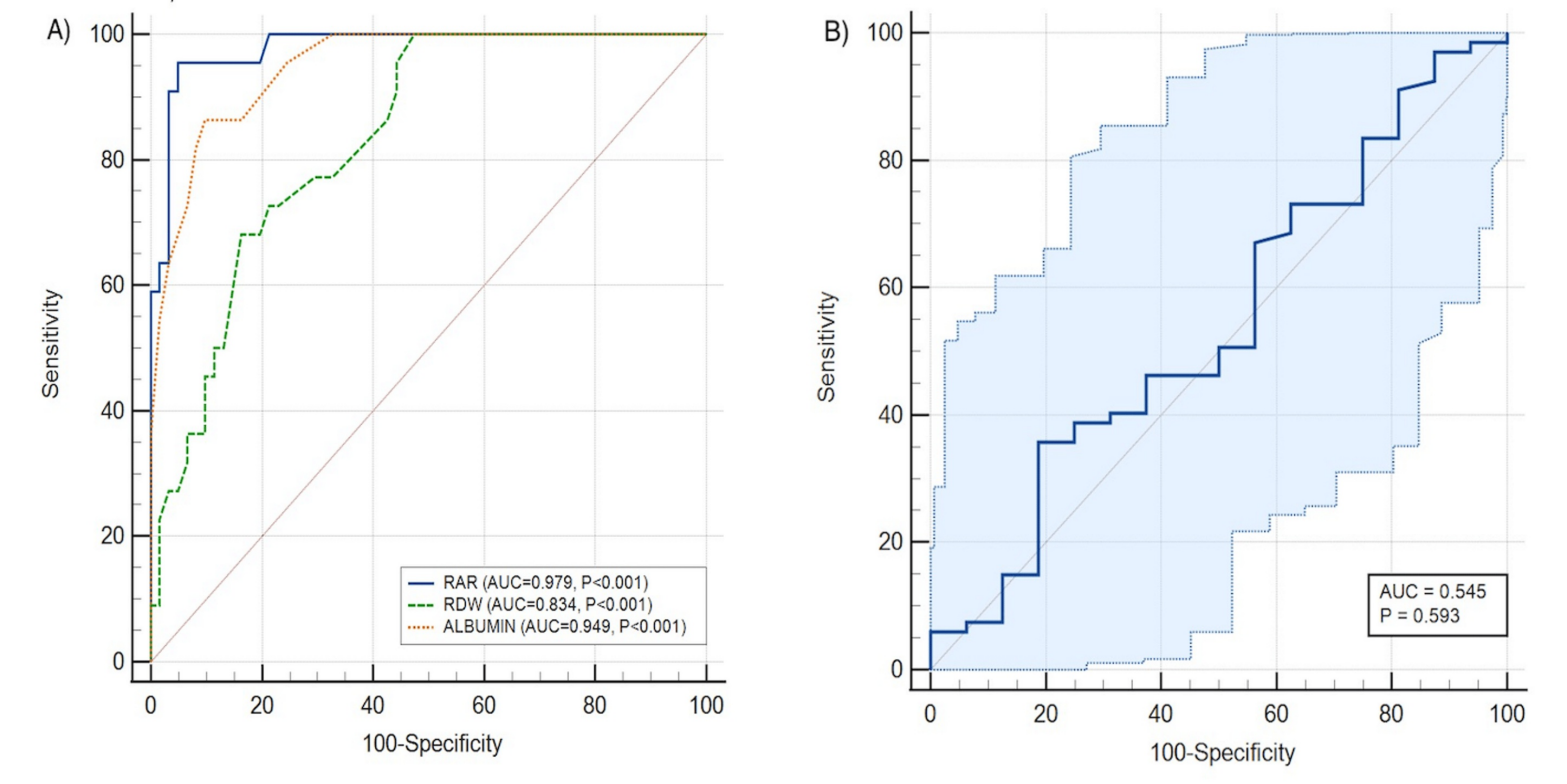
(A) ROC curves of baseline RAR, RDW, and albumin predicting 28-day mortality; (B) ROC curve of baseline RAR predicting NIV failure. RAR, red cell distribution width-to-albumin ratio; RDW, red cell distribution width; NIV, non-invasive ventilation; ROC, receiver operating characteristic

A baseline RAR threshold of >5.43%dL/g was identified as the optimal cutoff for predicting 28-day all-cause mortality. This cutoff demonstrated excellent predictive performance, with an AUROC of 0.979 (CI: 0.920 to 0.998; *P* < 0.001), sensitivity of 95.45%, specificity of 95.08%, and an accuracy of 95.18% (Figure 2A). On secondary analysis, a baseline RDW and albumin cutoff value of >13.6% and ≤ 2.7 g/dL predicted the 28-day all-cause mortality at an AUROC of 0.834 (CI: 0.736-0.907, sensitivity: 100%, specificity: 52.46%, accuracy: 65.06%, *P* < 0.001) and 0.949 (CI: 0.878-0.985, sensitivity: 86.36%, specificity: 90.16%, accuracy: 89.16%, *P *< 0.001), respectively (Figure 2A). In contrast, a baseline RAR cutoff of >5.107%dL/g showed poor predictive ability for NIV failure, with an AUROC of 0.545 (CI: 0.432-0.655; *P *= 0.59), sensitivity of 35.82%, specificity of 81.25%, and an accuracy of 44.58% (Figure 2B).

**Figure 3:**
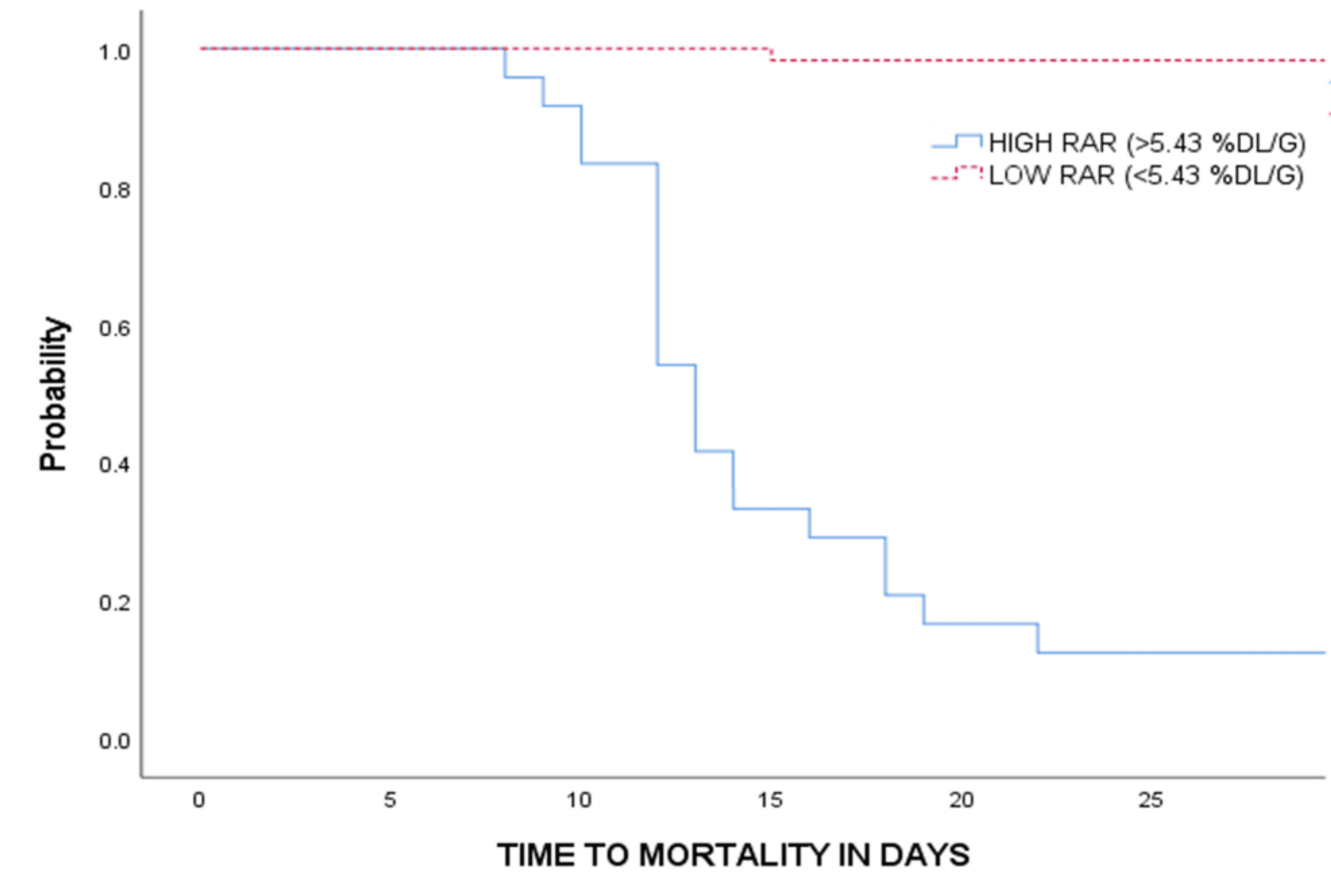
Kaplan-Meier curve of 28-day mortality stratified by high baseline RAR (>5.43%dL/g). RAR, red cell distribution width-to-albumin ratio

Patients were stratified into two groups based on the RAR threshold of 5.43%dL/g: lower and higher RAR. Compared to the lower RAR group, patients in the higher RAR group were older, had higher serum lactate and clinical severity scores, including APACHE II, SOFA, and CCI, had the presence of coronary artery disease, and had lower weight, BMI, and baseline hemoglobin (Table 1). In terms of outcome, the higher RAR group experienced significantly worse clinical results, including fewer mechanical ventilation (MV)-free days, organ failure-free days, vasopressor-free days, and days alive outside the hospital, higher requirement of invasive MV, incidence of new-onset bacteremia/fungemia; RRT requirement, blood transfusions, and 28-day mortality (Table 2). Notably, 21 out of 24 patients (87.5%) with high RAR died within 28 days, compared to just 1 out of 59 patients (1.69%) in the low RAR group. Kaplan-Meier survival analysis confirmed significantly better survival in the low RAR group (log-rank χ² = 86.184, *P* < 0.001) (Figure 3).

**Table 2:** Comparison of outcome variables between higher baseline RAR group versus lower baseline RAR group. Group 1: baseline RAR >5.43%dL/g, Group 2: baseline RAR <5.43%dL/g ***Significant at *P *< 0.05. ^€^Chi-square test. ^£^Wilcoxon-Mann-Whitney U test. Continuous non-parametric data presented as median (interquartile range), and categorical variables presented as frequency (percentage). NIV, noninvasive ventilation; MV, mechanical ventilation; ICU, intensive care unit; RRT, renal replacement therapy

Outcomes	Total (*n* = 83)	Group 1 (*n* = 24)	Group 2 (*n* = 59)	*P*-value
Time to NIV Failure (*n* = 67)	1 (1-2)	1 (1-1)	1 (1-2)	0.097^£^
MV free days (*n* = 83)***	16 (0-20)	0 (0-0)	18 (16-20)	<0.001^£^
Organ Failure free days (*n* = 83)***	18 (0-21)	0 (0-0)	20 (16-22)	<0.001^£^
Vasopressor-free days (*n* = 83)***	28 (24-28)	11 (8-22)	28 (28-28)	<0.001^£^
Time to ICU discharge (*n* = 61)	8 (7-12)	15 (5-16)	8 (7-12)	0.381^£^
Time to hospital discharge (*n* = 61)	10 (8-12.5)	18 (8-18)	10 (8-12)	0.172^£^
Days alive outside hospital (*n* = 61)***	18 (15.5-20)	0 (0-0)	18 (16-20)	<0.001^£^
Time to mortality (*n* = 22)	12.5 (12-15)	12 (12-15)	15 (15-15)	0.377^£^
Requirement of invasive MV***	71 (85.5%)	24 (100%)	47 (79.66%)	0.017^€^
NIV failure	67 (80.7%)	21 (87.5%)	46 (77.96%)	0.318^€^
Recurrence of invasive MV (*n* = 67)	4 (4.8%)	2 (9.52%)	2 (3.38%)	0.407^€^
New onset Bacteremia/Fungemia***	26 (31.3%)	18 (75%)	8 (13.55%)	<0.001^€^
Requirement of RRT***	16 (19.3%)	13 (54.16%)	3 (5.08%)	<0.001^€^
Requirement of blood transfusion***	9 (10.8%)	9 (37.5%)	0 (0%)	<0.001^€^
28-day mortality***	22 (26.5%)	21 (87.5%)	1 (1.69%)	<0.001^€^

Univariate analysis using log-rank/Cox PH identified several variables significantly associated with 28-day mortality, including age (*P* = 0.006), history of stroke (*P* = 0.004), coronary artery disease (*P* = 0.011), lactate (*P* < 0.001), hemoglobin (*P* = 0.002), hematocrit (*P* = 0.036), RDW (*P* < 0.001), albumin (*P* < 0.001), RAR (*P* < 0.001), APACHE II score (*P* < 0.001), SOFA score (*P* < 0.001), CCI score (*P* = 0.007) (Table 3). No significant collinearity was observed among variables included in the multivariate model (Variance Inflation Factor (VIF) < 2, tolerance > 0.2). On multivariable analysis, only baseline lactate (*P* = 0.011) and RAR (*P* = 0.008) remained statistically significant, indicating their independent predictive value for 28-day mortality in patients with ECOPD (Table 3). The proportional hazards assumption was evaluated using the Grambsch-Therneau test based on scaled Schoenfeld residuals. The tests were not significant at an for any univariate Cox regression analysis (alpha = 0.05). For Multivariate cox-regression, the global test result was non-significant (P = 0.21), thereby supporting the proportional hazards assumption. Furthermore, all individual predictors except for SOFA demonstrated a statistically non-significant result in the Grambsch-Therneau test. Even for SOFA, visual inspection of Schoenfeld residual plots did not reveal a systematic time-dependent effect.

**Table 3:** Univariate and multivariate Cox proportional hazards regression analysis of predictors of 28-day mortality in patients admitted with ECOPD. ***Significant at *P *< 0.05. RDW, red cell distribution width; RAR, red cell distribution width-to-albumin ratio; APACHE, acute physiological and chronic health evaluation; SOFA, sequential assessment of organ failure; CCI, Charlson comorbidity index

Parameter	Univariate analysis		Multivariate analysis	
	HR (95% CI)	*P*-value	HR (95% CI)	*P*-value
Age (Years)***	1.06 (1.02-1.10)	0.006	0.99 (0.92-1.06)	0.747
Stroke***	26.91 (2.80-258.75)	0.004	2.61 (0.22-30.71)	0.447
Coronary artery disease***	4.11 (1.38-12.22)	0.011	2.22 (0.42-11.74)	0.346
Lactate***	11.45 (4.59-28.55)	<0.001	5.52 (1.48-20.54)	0.011
Hemoglobin***	0.66 (0.51-0.86)	0.002	0.93 (0.66-1.31)	0.688
RDW***	1.39 (1.20-1.60)	<0.001		
Albumin***	0.04 (0.02-0.13)	<0.001		
RAR***	2.50 (1.90-3.29)	<0.001	2.11 (1.21-3.69)	0.008
APACHE II score***	1.20 (1.12-1.29)	<0.001	1.01 (0.81-1.27)	0.926
SOFA score***	1.44 (1.26-1.65)	<0.001	1.31 (0.96-1.80)	0.091
CCI score***	1.42 (1.09-1.84)	0.007	0.59 (0.33-1.07)	0.081

## Discussion

We observed that a baseline RAR cutoff of >5.43%dL/g had excellent predictive performance for 28-day all-cause mortality, with an AUC of 0.979, sensitivity of 95.45%, specificity of 95.08%, and an overall accuracy of 95.18% in patients admitted with ECOPD. Secondary analysis showed that baseline RAR had a better diagnostic profile to predict 28-day all-cause mortality compared to RDW and albumin individually. These results are highly encouraging and suggest that RAR is a strong prognostic marker in ECOPD.

Our findings align with and build upon prior research. In a retrospective ICU study, Qiu et al. reported an RAR cutoff of >5.315%dL/g to predict hospital mortality in patients with ECOPD, with moderate diagnostic performance (AUC 0.706) [5]. Although the threshold identified was similar to ours, the diagnostic profile in our prospective cohort was significantly stronger. In a retrospective design, Jeong et al. observed that RAR >5.73%dL/g predicted 28-day mortality in patients with pneumonia (AUC 0.694), and Yoo et al. reported a cutoff of >4.59%dL/g associated with 60-day mortality in patients with acute respiratory distress syndrome (ARDS) (AUC 0.681) [10,11]. Similar thresholds across diverse respiratory conditions support the broad applicability of RAR as a biomarker of systemic inflammation and adverse outcomes in acute respiratory illnesses.

A similar association between elevated RAR and increased mortality has also been observed in other critical illnesses. Zhou et al. demonstrated that high RAR was significantly associated with both 90-day and 365-day mortality in diabetic ketoacidosis [12]. Similarly, Liu et al. reported that RAR is an independent predictor of 30-day mortality in acute ischemic stroke [13]. Jian et al. found that an RAR cutoff >4.776%dL/g (AUC 0.738) was significantly associated with in-hospital all-cause mortality among ICU patients with acute myocardial infarction and that RAR was a more accurate predictor than RDW or albumin alone [14]. Our findings extend these observations, further supporting RAR as a negative prognostic marker across a broad spectrum of acute illnesses, including COPD.

Stratified analysis revealed that patients with high baseline RAR were older, had lower BMI, higher lactate, and worse clinical severity scores (APACHE II, SOFA), consistent with more advanced disease. Additionally, this group had a higher prevalence of coronary artery disease. These findings are in line with Qiu et al., who reported worse baseline physiologic and clinical severity markers in patients with elevated RAR [5].

We observed that mortality was significantly higher in the high RAR group: 87.5% vs. 1.69% in the low RAR group, demonstrating a strong association (Cramer's V: 0.88). Kaplan-Meier survival analysis further confirmed significantly improved survival in the lower RAR group over the 28 days (log-rank χ² = 86.184; *P* < 0.001). These results echo the observations by Qiu et al. in ECOPD and others in ARDS and pneumonia, over varied time horizons, reinforcing the prognostic role of RAR in critical care settings [5,10,11].

However, the predictive value of RAR for NIV failure in our cohort was poor (AUC 0.545; *P* = 0.59). This is likely due to the high proportion of NIV failures (80.7%) and the small sample size, which may have limited statistical power. Despite its low accuracy, the observed cutoff of >5.107%dL/g may have exploratory relevance in identifying high-risk patients but is inadequate for use as an independent predictor of NIV failure.

In terms of outcomes, the high RAR group had significantly fewer MV-free, organ failure-free, and vasopressor-free days, and a higher need for invasive mechanical ventilation, renal replacement therapy, and blood transfusions. They were also more likely to develop new-onset bacteremia or fungemia. Interestingly, there was no significant difference in ICU or hospital stay duration between RAR groups, an observation also made by Qiu et al. after adjusting for confounders [5]. However, past literature indicates that both elevated RDW and hypoalbuminemia are independently associated with prolonged hospitalizations in ECOPD, suggesting that dynamic trends in these markers may still carry prognostic relevance [15,16].

This study has some limitations. First, the heterogeneity in the etiology of ECOPD (infective vs. non-infective) may have influenced RAR levels, and subgroup analysis by etiology was not performed due to the limitation of sample size. Second, we assessed only baseline values of RDW and albumin; dynamic changes over time may offer additional prognostic insight. Third, the single-center design and relatively small sample size may limit generalizability, and selection bias is possible due to the tertiary care setting and late referrals, potentially introducing Berkson’s bias. Fourth, the possibility of overfitting may have resulted in an overestimation of the discriminatory performance of RAR, which could limit its external validity or generalizability. Nevertheless, the strong and consistent association between elevated RAR and 28-day mortality reinforces its potential as a prognostic marker.

## Conclusions

In conclusion, baseline RAR demonstrated good discriminatory performance for predicting 28-day all-cause mortality in patients with ECOPD and outperformed RDW and albumin individually. A RAR cutoff >5.43%dL/g was independently associated with higher mortality and greater clinical severity. While RAR showed limited utility in predicting NIV failure, these findings suggest that RAR may serve as a useful prognostic marker in ECOPD. External validation in larger, multicenter studies is required before routine clinical application.
